# TLR2 and TLR4 as Potential Biomarkers of Environmental Particulate Matter Exposed Human Myeloid Dendritic Cells

**Published:** 2007-05-30

**Authors:** Marc A. Williams, Chris Cheadle, Tonya Watkins, Anitaben Tailor, Smruti Killedar, Patrick Breysse, Kathleen C. Barnes, Steve N. Georas

**Affiliations:** 1 University of Rochester School of Medicine and Dentistry, Division of Pulmonary and Critical Care Medicine, Rochester, New York, U.S.A; 2 Johns Hopkins University School of Medicine, Division of Allergy and Clinical Immunology, Baltimore, Maryland, U.S.A

**Keywords:** Toll-Like Receptors, Inflammation, Dendritic Cell, Pollution, Particulate Matter

## Abstract

In many subjects who are genetically susceptible to asthma, exposure to environmental stimuli may exacerbate their condition. However, it is unknown how the expression and function of a family of pattern-recognition receptors called toll-like receptors (TLR) are affected by exposure to particulate pollution. TLRs serve a critical function in alerting the immune system of tissue damage or infection—the so-called “danger signals”. We are interested in the role that TLRs play in directing appropriate responses by innate immunity, particularly dendritic cells (DC), after exposing them to particulate pollution. Dendritic cells serve a pivotal role in directing host immunity. Thus, we hypothesized that alterations in TLR expression could be further explored as potential biomarkers of effect related to DC exposure to particulate pollution. We show some preliminary data that indicates that inhaled particulate pollution acts directly on DC by down-regulating TLR expression and altering the activation state of DC. While further studies are warranted, we suggest that alterations in TLR2 and TLR4 expression should be explored as potential biomarkers of DC exposure to environmental particulate pollution.

## Introduction

Dendritic cells (DC) are the sentinels of the innate immune system, and evolved to translate diverse environmental cues into signals that could rapidly activate the adaptive arm of immunity and particularly the CD4+ T lymphocytes (1–4). Immature DC are specialized for taking up exogenous antigen by mechanisms that include endocytosis (1, 2), yet they exhibit a poor ability to stimulate T cells unless activated (3, 4). Immature DC capture antigen and migrate to the draining lymph nodes. It is thought that maturation takes place during their migration where DC are now defined as potent stimulators of T cell proliferation with a suppressed ability to take up exogenous antigen.

In 1989, David Strachan proposed the “hygiene hypothesis” after his group found that hay fever was less prevalent in children from large families (5). This hypothesis associates exposures to microbial particles or their by-products and the subsequent development of allergic inflammation and disease. The association is a function of or dependent on the interactions of the environment with the immune system, the time of exposure to microbial agents (i.e. childhood or adolescent exposures) and the genetic susceptibility of the host to allergic diseases. The hypothesis implicates a protective role from developing allergic diseases in individuals that have had prior exposure to acute or non-disease causing infections with viruses and bacteria or exposure of the individual to environmental particulates that are rich in non-viable microbial fragments or by-products. The hygiene hypothesis therefore attempts to relate the contribution that microbial exposures in the environment may play in modulating the innate and adaptive immune response in either promoting or by contrast protecting the human subject form developing allergic diseases such as asthma.

Epidemiological data and clinical studies have shown that exposing children to others in family or day care facilities reduces the risk of allergic disease and thus of developing allergic asthma or hay fever in later childhood (6–8). In addition, early life exposure to farm animals or paradoxically domestic animals also confers protection or at least dampens the risk of acquiring allergic diseases (9–11). These exposures point to a hygiene-related protective affect that although controversial is supported by the evidence. That said the precise nature of the factors that contribute to the protective effect remains obscure.

Although DC are poised to respond to inhaled pollutants including ambient particulate matter (PM), surprisingly little is known about whether and how PM affects the function of DC. Immature DC respond aggressively to danger signals (e.g. endotoxin) such that a primary immune response is appropriately induced (11). Danger signals are transduced by pattern recognition receptors that include the toll-like receptor (TLR) family (12). Occupation of the TLR by ligand (e.g. LPS binding to TLR4) instructs DC to mature (13). DC can also be activated by T-cell dependent signals such as the ligand for the co-stimulatory receptor CD40 (CD40 ligand, or CD40L). DC maturation is defined by enhanced cell surface expression of co-stimulatory molecules, reduced endocytosis, enhanced antigen presentation, stimulation of T cell proliferation and increased cytokine production (1–4, 14).

In the current study, we show that expression of TLRs by dendritic cells and the associated signal transduction proteins are dysregulated following exposure to PM. We speculate that alterations in expression of both TLR2 and TLR4 could be adopted as potential biomarkers of DC exposure to environmental particulate matter (PM).

## Materials and Methods

### Reagents

For DC culture, we used the recombinant human cytokines interleukin-4 (IL-4) and granulocyte-macrophage colony-stimulating factor (GM-CSF), both from Peprotech Inc. (Rocky Hill, New Jersey). Lipopolysaccharide (LPS, *E. coli* 0.55:B5) was obtained from Sigma-Aldrich (St. Louis, MO). Recombinant human CD40-Ligand (CD40L trimer) was a gift from Dr John McDyer, Johns Hopkins University. For flow ccytometry FITC-conjugated fluorochromes were; IgG1-κ (isotypic control, clone MOPC-21), HLA-DR (IgG2a-κ, clone G46-6), and PE-conjugated fluorochromes were; IgG1-κ (isotypic control, clone MOPC-21), IgG2a-κ (isotypic control, clone G155-178), CD80 (IgG1, clone L307.4), CD83 (IgG1, clone HB15e) and CD86 (IgG1, clone FUN-1) all from Pharmingen (San Diego, CA). The following PE-conjugated fluorochromes were obtained from Santa Cruz Inc. (Santa Cruz, CA); TLR-2 (IgG2a, clone TL2.3) and TLR4 (IgG2a, clone HTA125).

### Generation of CD14+ monocyte-derived immature DC

DC were generated from CD14+ peripheral blood monocytes using cytokine-driven propagation of dendritic cell precursors from non-allergic, non-asthmatic, non-smoking and apparently normal healthy adult subjects (2). Monocytes were enriched from venous blood under protocols that were approved by the Institutional Review Boards and obtaining informed consent from donor subjects. Monocytes were enriched by positive selection and magnetic activated cell sorting of CD14+ cells (MACS, Miltenyi Biotec, Auburn, CA). Enriched monocytes were seeded into 10 cm^2^ culture dishes at a density of 5.0 × 10^5^ cells/ml in a total volume of 8 ml of culture medium in a 5% CO_2_/95% air and fully humidified atmosphere. Monocyte-derived DCs were cultured continuously for 10 days in an RPMI-1640 (Dutch modification) base culture medium (GIBCO/BRL) supplemented with 8% v/v FBS, 2 mM L-glutamine, 1 mM sodium pyruvate, 1% v/v non-essential amino acids, 2.5 μg/mL gentamicin sulfate, 20 mM HEPES buffer (all from (GIBCO-BRL) and 5 × 10^−4^ M 2-mercaptoethanol (Sigma). Cultures of DC were pulsed with rHuGM-CSF at 25 ng/ml and rHuIL-4 at 20 ng/mL on days 0, 2, 5, 8 to permit DC development. Dendritic cells were harvested at day 10 for in vitro experiments.

### Ambient particulate matter

Ambient PM was collected from ambient outdoor air in Baltimore City, MD in the spring of 2001 (April-June) using a high-volume cyclone collector with a theoretical cut point of 0.85 μm aerodynamic diameter. The particle size distribution was determined using phase contrast optical microscopy. The count median diameter of the particle size distribution was 1.8 μm. Collected APM was pooled, refrigerated until use and protected from light. Prior to use 10 mg/ml of APM was suspended in 20 mM HEPES-buffered divalent cation-free PBS pH 7.4, vortexed at high-speed for 5 min and used immediately. The toxicity of APM was tested against human myeloid DC as well as murine bone-marrow-derived DC by monitoring trypan blue exclusion. After 48h of culture, the toxic dose of APM that induced 50% killing (TD50) was 660 μg/ml for human APM and 540 μg/ml for murine DC (data not shown). All subsequent experiments were done using APM titrated between 0.1 and 100 μg/ml. APM at 100 μg/ml (the highest dose used in our studies) was assayed for contaminating endotoxin levels using the Limulus Amebocyte Lysate QCL-1000 assay (Cambrex Inc., Walkersville, MD) and was less than 50 pg of endotoxin per 100 μg of APM. We conduced an elemental composition analysis of Baltimore PM by inductively coupled plasma mass-spectroscopy. We found that it is was rich in the following transition or heavy elements (16): Aluminum 9889.9 μg/g; Copper 5071.8 μg/g; Iron 23639 μg/g; Magnesium 5532.7 μg/g; Manganese 1106.1 μg/g; Titanium 2359.9 μg/g and Zinc 1641.2 μg/g. Other elements that were present at appreciable levels (100–250 μg/g mass range) included chromium, lead, strontium and vanadium.

### Stimulation of DC cultures with ambient particulate matter

CD14+ monocyte-derived DC were harvested at day 10 of culture and seeded into 12-well culture dishes in duplicate at a density of 2.0 × 106 cells/well in a total volume 2.0 mL. In most experiments DC were stimulated for 48h with the following agents: LPS (a classical TLR agonist) at 5000 pg/mL, CD40 ligand (a TLR-independent agonist, 50 ng/mL) and titrated doses of APM at 100, 10, 1 and 0.1 μg/mL as well as doses of purified LPS that were present in the stated titrated doses of APM. For example, in 100 μg/mL of APM we found equivalently 50 pg/mL of endotoxin as determined by the Limulus Amebocyte Lysate QCL-1000 assay (Cambrex Inc., Walkersville, MD). Additional controls included LPS (a TLR4 agonist, *E. coli* serotype 055:B5 derived endotoxin) used at a concentration of 100 ng/mL and CD40-ligand (CD40L, a non TLR-agonist) was used at 50 ng/mL. Cultures were incubated for 48h in a 5% CO_2_/95% air and a fully humidified incubator.

### Phenotypic analysis of cell-surface and toll-like receptor expression by flow cytometry

DC were prepared for flow cytometric analyses as described previously (2). After washing DC cultures twice in FACS buffer (divalent cation-free PBS, 20 mM HEPES, 2.5% v/v FBS, 0.02% w/v sodium azide), DC were pre-incubated with 5% v/v normal human AB serum to block non-specific staining of test monoclonal antibodies against Fc-gamma receptors, then washed twice in ice-cold FACS buffer. DC were subsequently stained with the appropriate antibodies as listed above. DC were aliquoted into labeled tubes at a density of 1 × 10^5^ cells in a volume of 100 μl and incubated with the respective isotype-matched irrelevant control antibody or the specific test monoclonal antibody for 30 min, on ice, in the dark. Antibodies were titrated for optimal concentrations in pilot experiments. Also, the same lots of all monoclonal antibodies were used throughout the study. Samples were washed twice in FACS buffer at 400 g for 6 min at 4 °C and post-fixed in 2% v/v paraformaldehyde fixative in FACS buffer prior to analysis.

Samples were analyzed immediately on a FACScaliber flow cytometer using CellQuest 3.1 software (Becton Dickinson). The instrument had a standard optical filter configuration with band pass filters of 530/30-nm and 585/44-nm for FL1 (FITC-conjugated antibodies) and FL2 (PE-conjugated antibodies) data acquisition respectively. The instrument was standardized prior to phenotypic analysis with Calibration beads (FluoroSpheres 6-Peak; Dako Cytomation). For the analysis of forward-angle light scatter, side-angle light scatter, and cell surface receptor expression data were acquired in real time. Data was recorded as geometric mean fluorescence intensity (MFI) and percent fluorescent positive cells. Prior to sample acquisition, the flow cytometer was cleaned with sequential washes of distilled water, 10% v/v hypochlorite and distilled water.

### Immunofluorescence microscopy

Dendritic cells were stimulated without or with APM at 100 μg/ml for 48h, washed twice in complete culture medium and resuspended in 100 μl of ice-cold FACS buffer and placed on ice. Cells were pre-incubated with 5% v/v normal human AB serum to block non-specific staining of test monoclonal antibodies against Fc-gamma receptors, then washed twice in ice-cold FACS buffer. DC were aliquoted into labeled tubes at a density of 1 × 10^5^ cells in a volume of 100 μl and incubated with the respective isotype-matched irrelevant control antibody or the specific anti-human TLR2 or TLR4 monoclonal antibody for 30 min, on ice, in the dark. Samples were washed twice in FACS buffer at 400 g for 6min at 4 °C and resuspended in 50 μl of FACS buffer prior to loading the sample onto a superfrost microscope slide and No. 02 coverslip. After the slides were prepared, we captured immunofluorescent microscopic images by photographing and saving them electronically at room temperature. This was done using a Carl Zeiss Axio-Observer Z.1 phase contrast/fluorescent microscope equipped with the Apotome Imaging System and a 100x objective lens under oil immersion (Carl Zeiss Microimaging Inc. Thornwood, NY).

### Quantitative real-time polymerase chain reaction (qRT-PCR) analysis

Reverse transcription was done on total RNA isolated from human DC. For this analysis, human monocyte-derived DC were propagated as described above and seeded into 6 well culture dishes at 2.5 × 10^6^ DC per ml in a total volume of 2 ml per well for a total DC density of 5 million cells per treatment. DC were treated with or without ambient urban (Baltimore City) particulate matter at 10 μg/ml for a period of 6h and at 37 °C in a fully humidified 5% CO_2_ in 95% air incubator. Following this incubation, DC were passed over 13.5% v/v iodixanol in PBS cell separation medium to separate DC from excess particulate matter by centrifugation at 600 g at 20 °C for 30 min with brake off and minimum acceleration. Interface DC, were harvested, washed in divalent cation-free PBS/20 mM HEPES pH 7.4 and processed with the Applied Biosystems (Foster City, CA) high-capacity cDNA archive first-strand synthesis system for RT-PCR according to the manufacturer’s protocol. We used this technique to assay for Toll-like receptor pathway RNA transcripts as well as inflammatory cytokine RNA transcripts. QRT-PCR was performed using the RT^2^ Profiler^™^ PCR array from SuperArray (Gaithersburg, MD). RT^2^ Profiler^™^ PCR arrays are designed for relative quantitative QRT-PCR based on SybrGreen detection and performed on a one sample/one plate 96-well format using primers for a preset list of genes corresponding to a particular biological pathway. We studied the human Toll-Like Receptor Signaling Pathway PCR array (APH-018) and the Human Inflammatory Cytokines & Receptors PCR Array (APH-011). In brief, cDNA volumes were adjusted to ~2.5 ml with SuperArray RT^2^ Real-Time SYBR Green/ROX PCR 2 × master mix (PA-012). Control reactions of no template, RNA only, and serial dilutions of template (for ACTB) were prepared separately then 25 μl of the cDNA mix was added to wells A1-H5. Next, 25 μl of the serial dilutions were added to wells H6-H10. In addition, 25 μl of “No RT” mix was added to well H11, and 25 μl of 1x master mix alone was added to well H12.

The PCR plate was sealed and spun at 400 g, for 4 min at 4 °C and QRT-PCR was performed on an Applied Biosystems 7300 RT-PCR System (Foster City, CA). ABI instrument settings included setting reporter dye as “SYBR” and setting passive reference as “ROX”. We deleted the UNG activation by highlighting and pressing delete, adding dissociation stage and changing sample volume to 25 μl. Relative gene expressions were calculated by using the 2^−ΔΔCt^ method, in which Ct indicates cycle threshold, the fractional cycle number where the fluorescent signal reaches detection threshold (15). The normalized ΔCt value of each sample is calculated using up to a total of 5 endogenous control genes (18S rRNA, HPRT1, RPL13A, GAPDH, and ACTB). Fold change values are presented as average fold change = 2^−(averageΔΔCt)^ for genes in treated relative to control samples.

### Biometry and statistical measurements

Data are expressed as mean ± 1 SD or 1 SEM as described. A minimum of n = 6 independent experiments were completed as described in the text. Comparisons between data were tested for significance using ANOVA, Student’s T-test and post-hoc correction according to the Bonferroni method. Statistical significance was set at an alpha value of at least p < 0.05 as indicated in the text.

## Results

### Modulation of the expression of function-associated molecules by DC

**Figure 1:**
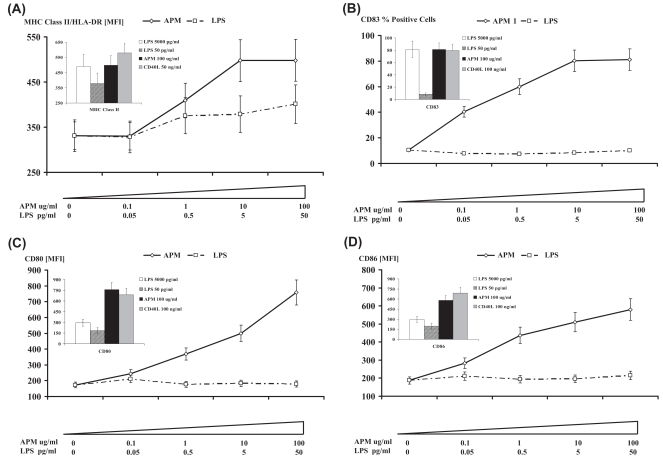
Flow cytometric quantitation of the functionally-important molecules MHC class II/HLA-DR (A), CD83 (B) CD80 (C) and CD86 (D) on the cell surface of resting and stimulated DC. DC were dose-dependently stimulated with APM and contaminating doses of LPS found in APM. In addition, we show the expression of these cell surface-expressed molecules on DC following their interaction with APM (100μg/ml), high-dose LPS (5000 pg/ml) and CD40L (50ng/ml) for 48 hours as indicated in the bar charts inset. Data is recorded as geometric mean fluorescence intensity (FI) units or percent fluorescent positive cells as appropriate ± SD. The levels of significance shown are: * P < 0.05 and ** P < 0.01 as compared with resting DC.

We compared the effects of DC exposure to APM with two known DC activators namely CD40L and LPS, representing T cell- and TLR-dependent pathways of DC activation, respectively (Figure 1). We used multi-parameter flow cytometry to determine the cell surface expression of functionally important molecules. These included MHC class II (HLA-DR), CD83, CD80 and CD86 on resting as well as activated DC following their interactions with APM. The expression of HLA-DR is an important molecule involved in the presentation of endogenously processed antigen to CD4 + T cells. The expression of HLA-DR by DC was enhanced following stimulation with CD40L or LPS as compared with resting DC (Figure 1a, P < 0.05). This is consistent with conventional maturation of DC. However, APM failed to alter HLA-DR percent positive expression on DC but it did enhance mean fluorescence intensity of HLA-DR on APM stimulated DC (Figure 1a). This was in contrast to previous studies of the effects of APM on CD34+ progenitor cell-derived DC where APM did not alter MHC class II expression at all (16). This most likely reflected the different cell sources and culture conditions used for studying the effects of APM on DC activation.

We also measured the level of expression of the maturation-associated marker CD83 (Figure 1b). As expected, CD83 expression was up-regulated when DC were stimulated with either CD40L or LPS (P < 0.01), an observation consistent with a conventional maturation program. However, APM provoked a marked and significant increase of CD83 expression (Figure 1b, P < 0.05), a feature of maturing and activated DC. The expression of both of the co-stimulatory molecules CD80 and CD86 were enhanced following stimulation of DC with CD40L or LPS as compared with resting DC (Figure 1c and d, P < 0.01). In addition, expression of both CD80 and CD86 were enhanced on DC that were exposed to APM as compared with resting DC (Figure 1c and d, P < 0.01), as previously reported (25). Enhanced expression of CD80 and CD86 by APM stimulated DC is consistent with conventional maturation of activated DC.

### Toll-like receptors TLR2 and TLR4 are markedly down-regulated by APM stimulated DC

**Figure 2:**
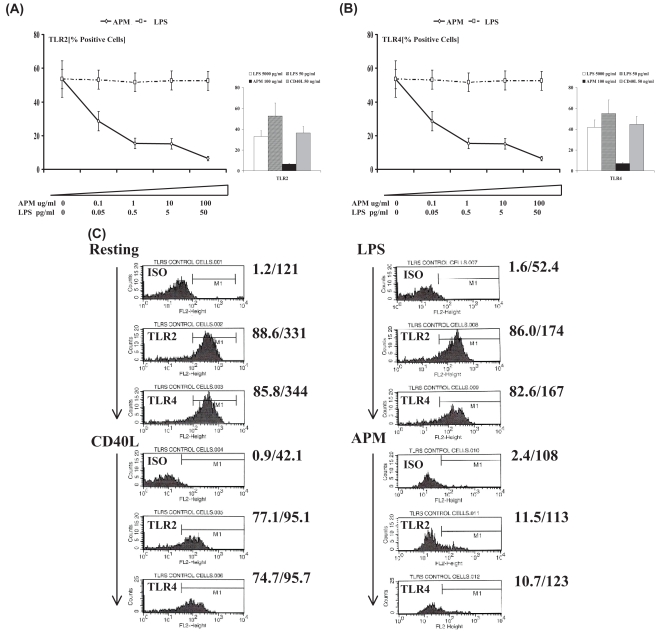
Flow cytometric quantitation of the cell-surface expression of (A) TLR2 and (B) TLR4 for resting and stimulated DC as in Figure 1. Data is recorded as geometric mean fluorescence intensity (FI) units ± SD. The levels of significance shown are: ** P < 0.01 or as *** P < 0.001 as compared with resting DC. In addition, we show the expression of TLRs following their interaction with APM (100μg/ml), high-dose LPS (100ng/ml) and CD40L (50ng/ml) for 48 hours as indicated in these original flow cytometric histograms (C).

**Figure 3:**
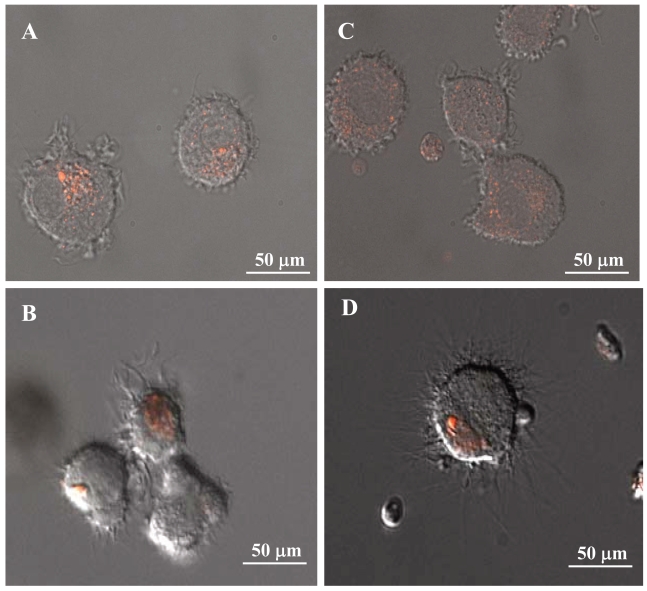
Immunoflourescent microscopic images of TLR2 (A and B) and TLR4 (C and D) expression by resting (A and C) and APM stimulated (B and D) dendritic cells. Note the rather focused expression of TLR2 proximal to the nucleus (A) while the expression of TLR4 shows a more peripheral and concentric pattern of expression (C). The expression of both TLR2 and TLR4 are lost following exposure to APM (B and D).

Toll-like receptors are important in directing the recognition of microbial and signatures of host tissue damage signals by DC and thereby initiating immune responses to danger signals such as infection or cell death. Thus, we asked the question of how the expression of these receptors may be modulated by dose-dependently stimulating DC with APM as compared with classical TLR signals such as that provided by LPS (Figure 2). We found that TLR2 expression by DC was down-regulated following stimulation with high-dose LPS (P < 0.001, Figure 2a and c). By contrast, DC that were exposed to APM exhibited a dramatic down-regulation of TLR2 expression (P < 0.001, Figure 2a and c), as previously reported (25). Even at the lowest concentration of APM tested (0.1μg/mL), we observed marked suppression in TLR-2 expression (P < 0.01). The expression of TLR4 (Figure 2b and c) followed the same pattern of response to LPS and APM as was observed for TLR2. This would suggest that the functional expression of two important pattern-recognition receptors is lost. In addition, our immunofluorescent analysis of TLR expression by DC was concordant with the above observations of diminished TLR2 and TLR4 expression by DC in response to APM stimulation (Figure 3). The pattern of fluorescence present on the cell surface of DC indicated that TLR2 and TLR4 expression is lost by DC that were exposed to environmental particulate matter (Figure 3). Expression of TLR2 by DC before (Figure 3a) and after (Figure 3b) stimulation with APM is shown and similar microscopic images are shown for TLR4 (Figure 3c and d). In both cases, non-stimulated DC exhibit widespread expression of TLR2 and TLR4 while expression of both of these pattern-recognition receptors was significantly less in DC that had been stimulated with APM (Figure 3b and d) as compared with resting non-stimulated DC (Figure 3a and c).

### QRT-PCR analysis of toll-like receptor and inflammatory cytokine gene sets

**Figure 4:**
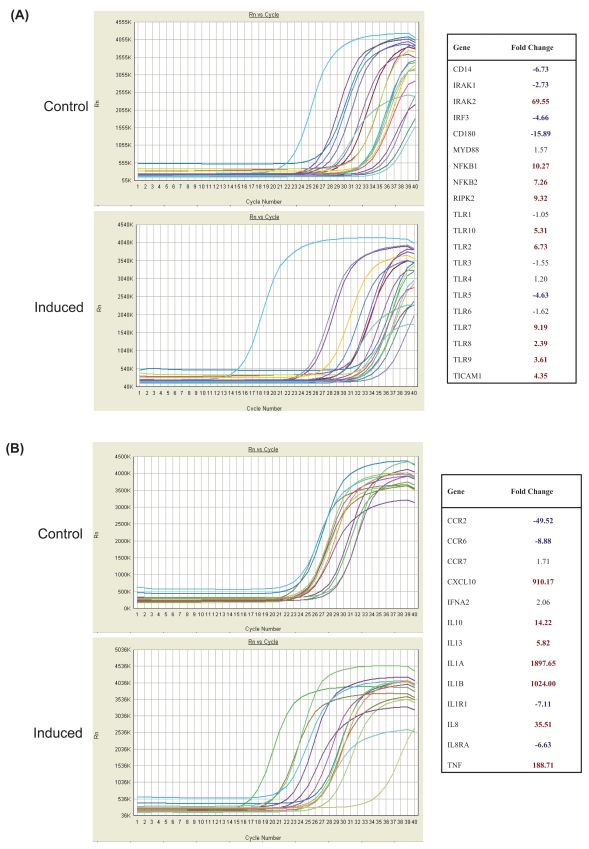
This data shows linear views of the signal amplification plots generated by real time PCR for selected subsets of genes. RNA samples were tested by RT-PCR using Sybr Green I dye and PCR-primer formats arrayed on 96-well micro-titer plates (SuperArray RT-Profiler). An entire panel of genes corresponding either to the Toll-like Receptors and their associated pathway genes (A) or inflammatory cytokines, their receptors, and associated pathway genes (B) were tested. The methodological approach is described in the text.

At the protein level, surface expression of both TLR2 and TLR4 were down-regulated in response to stimulation by ambient PM. Thus, we next wished to investigate the transcriptional expression of toll-like receptors and toll-like receptor signaling proteins (Figure 4) following stimulation of DC with or without ambient PM as compared with the transcriptional expression of a focused array of inflammatory cytokine gene sets (Figure 4). We obtained highly informative and detailed information with regard the regulation of TLR and signaling transcripts. Figure 4 (a, b) shows linear views of the signal amplification plots generated by real time PCR for selected subsets of genes. RNA samples were tested by RT-PCR using Sybr Green I dye and PCR-primer formats arrayed on 96-well micro-titer plates (SuperArray RT-Profiler).

An entire panel of genes corresponding either to the Toll-like Receptors and their associated pathway genes (Fig 4a) or inflammatory cytokines, their receptors, and associated pathway genes (Fig 4b) were tested. A profile shifted to the left following induction indicates an increase in gene expression (and a positive fold change as indicated in the accompanying data table) while a decrease in gene expression is indicated by a profile shift to the right (and a negative fold change in the accompanying data table). A single RT-PCR cycle represents a doubling of DNA target so that, for example, a three cycle difference (at half-maximum) between two samples for a given gene (ΔCt=3) represents 2^3^ difference in gene expression or an eight-fold increase or decrease in the original starting amounts of mRNA for that given gene in the test samples.

**Figure 5:**
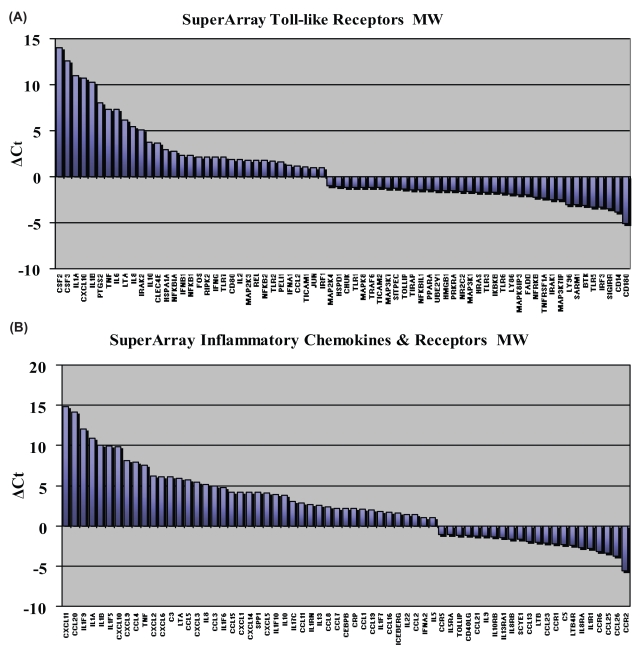
This figure reports out directly the ΔCt values for all genes which showed a 2-fold or greater change in gene expression between control and induced samples as measured either on the Toll-like Receptor SuperArrays (A) or for the Inflammatory Cytokine SuperArrays (B). Regulated genes on each array are shown rank-ordered from the largest positive to the largest negative change.

Figure 5 (a and b) reports out directly the ΔCt values for all genes which showed a 2-fold or greater change in gene expression between control and induced samples as measured either on the Toll-like Receptor SuperArrays (Figure 5a) or for the Inflammatory Cytokine SuperArrays (Figure 5b). Regulated genes on each array are shown rank-ordered from the largest positive to the largest negative change. We found that the expression of TLR transcripts were coordinately regulated with particular TLRs exhibiting enhanced expression and others showing down-regulated expression following interaction of DC with APM (Figure 4a and 5a). In particular, we noted modest down-regulation of mRNA encoding TLR1, TLR3, and TLR6 and remarkable down-regulation in the expression of TLR5 and CD180 (RP105). By contrast, TLR2 and TLR10 gene expression was upregulated as were the endosomal expressed receptors TLR7-9 while the expression of TLR4 was unaltered. Taken together with the flow cytometry analyses, these data indicate that TLR surface expression and gene expression do not always correlate: further studies will be needed to uncover the molecular mechanisms for this discrepancy.

Similarly, we found that the RNA transcript expression of TLR signaling and adaptor proteins followed a pattern of enhanced or suppressed expression after DC were exposed to APM (Figure 4a and 5a). For example, the putative endogenous ligand of TLR2 and TLR4 signaling was markedly down-regulated by APM-exposed DC as were the LPS co-receptor CD14, the TLR4 co-localizing adaptor protein MD2, SIGIRR which serves an important immune suppressive role in attenuating TLR2 and TLR4 signaling, IRF3 which is involved in the MyD88-independent signaling pathway and the expression of IRAK1 which serves an essential role in signaling through TLR7 and TLR9 (Figure 4a and 5a). By contrast, we also found that NFkappaB1p50 and NF-kappaB2p52 were markedly down-regulated following stimulation with APM, as was the expression of IRAK2, RIPK2/RICK and TICAM1 or TRIF.

We also observed that the RNA transcripts for IL-6, IL-12 and TNF-alpha were dramatically enhanced following stimulation with PM (Figure 4b and 5b). In addition, the RNA transcript expression of IL-1alpha, IL-1-beta, IL-10 and the IFN-alpha, -beta and –gamma were all markedly enhanced following exposure of DC to PM (Figure 4b and 5b). This data is consistent with a pro-inflammatory state of DC. We also noted that the myeloid hematopoietic growth factors G-CSF and GM-CSF were similarly augmented as was the functionally important chemokine CXCL10. Consistent with DC maturation, we observed that APM stimulated DC exhibited down-regulated RNA transcript expression of CCR6 and gain in expression of CCR7 (Figure 4b and 5b). The observations made for the regulation of the expression of inflammatory cytokine transcripts was concordant with some of the observations made above for cytokine secretion by DC. For example, we found that PM stimulated DC secreted substantially more GM-CSF, IL-6, IL-12 and TNF-alpha compared with unstimulated DC (25, and data not shown).

## Discussion

A major concern in environmental health is the global increase in ambient air pollution particularly in urban areas with high-density traffic-related pollution. However, the potential links between exposure to particulate matter air pollution and immune dysregulation are under-appreciated, poorly understood and warrant detailed study. There is a growing body of literature indicating that inhaled PM derived from industrial point sources and mobile sources in cities and surrounding urban areas contribute to the reported increases in the incidence of asthma, allergic conditions, respiratory infections, pulmonary infections and mortality in the infant and adult populations (17–21). Furthermore, it is apparent that different types of air pollution can have qualitatively different effects on human health (19). The National Research Council’s (NRC) Committee on Research Priorities for Airborne Particulate Matter (NRC 2004) identified understanding mechanism of PM injury and developing a better idea of the characteristics of PM that modulate toxicity as high priority research areas.

Dendritic cells are the key sentinels of the innate immune system that evolved to rapidly translate diverse environmental cues into signals that activate adaptive immunity following their interactions with pathogenic microorganisms or environmental particulates at the mucosal interface (22, 23). Moreover, the identification and characterization of different human DC subpopulations in the lung indicates to us their importance as key antigen-presenting cells in an appropriate anatomical setting to respond to inhaled particulate matter (24). Since respiratory tract DC are rapidly derived from circulating precursors (4, 25), our experiments provide a reasonable approximation of how DC and ambient environmental pollution particulates interact in vivo. DCs are densely distributed throughout the airways and respiratory epithelium, and may even directly sample the bronchial lumen (25). Therefore, one might expect that DC would be among the first cells to interact with and respond to inhaled particulate pollution.

We have previously shown that airborne particulate matter dysregulates the production of inflammatory cytokines by human dendritic cells as well as the interaction of DC with naïve CD4+ T cells (16). Further, we have previously provided a detailed elemental composition analysis of the collected Baltimore City ambient particulate matter using inductively coupled plasma mass spectroscopy (16). In this report, particulate matter-exposed DC secreted less IL-12 and IL-6 but exhibited increased secretion of IL-18 and IL-10 as compared with LPS stimulated DC. One of the most important observations from this work was a Th2-like pattern of cytokine production seen in co-cultures of particulate matter stimulated DC and alloreactive naïve CD4+ T cells where the IL-13 to IFN-γ ratio was reversed. This contrasted with the Th1 polarizing effects of high-dose LPS on DC. We reported for the first time that environmental particulate matter exposed DC directed a complex Th1/Th2-like pattern of T cell activation by mechanisms that involved a non-classical activation of DC. These data have significant clinical implications. Foremost among these are that inhaled PM can act directly on DC stimulating a danger signal to direct a pro-allergic pattern of innate immune activation.

We found that, in the resting state, myeloid DC expressed relatively high levels of both cell-surface TLR2 and TLR4. Our observations were concordant with another study that showed under resting conditions, human myeloid DC express mRNA transcripts for TLR2 and TLR4 (26, 27). Moroever, it was found that by flow cytometric analyses, human myeloid DC expressed high levels of TLR2 and moderate levels of TLR4 (28). In our current study, we found that when myeloid DC were exposed to particulate airborne pollution, the expression of both TLR2 and TLR4 were significantly down-regulated. Both TLR2 and TLR4 serve critical functions in linking innate and adaptive immunity. Both of these receptors recognize microbial products following which immune responses are activated to a broad array of microbial challenges (29,30).

In our elemental analyses, we discovered a number of transition and heavy metals (16) in the ambient urban particulate matter sample that may have induced some interesting immunological effects against DC. However, we await formal demonstration of the specific effects of those metals on the immunological activation of DC. Prior reports have shown adverse effects of metals commonly found in atmospheric pollution and ambient particulate matter on the immunological function of dendritic cells and other cells (31–34). A number of these elements promote an allergic response (31). For example, monocyte-derived DC have been exposed to the contact allergens nickel sulfate, cobalt chloride, palladium chloride, copper sulfate, chromium III chloride and potassium dichromate (31). In this study, it was found that only nickel sulfate, cobalt chloride and copper sulfate augmented the expression of the DC maturation markers CD83 and CD86 but that all of the allergic compounds induced secretion of CXCL8 (31). Others have shown that the contact allergen nickel sulfate altered a broad range of genes and biological processes in human stem cell-derived DC (32). In particular, molecules and cell-surface receptors involved in DC maturation and antigen uptake/processing such as DEC205, DC LAMP and CCR7 were enhanced after exposure of DC to nickel sulfate (32).

One of the metals that were found to be coalesced at relatively high levels in our study was chromium. It has been previously shown that inhaled hexavalent chromium can induce pulmonary diseases and lung cancer (33). In the A549 lung epithelial cell line model, nontoxic doses of chromium enhanced production of reactive oxygen intermediates and preferentially activated c-Jun N-terminal kinase (JNK) and Src family kinases independently of reactive oxygen intermediate production (33). Although platinum *per se* was not an element that stood out on our “hit list” of highly prevalent elements, other have shown that particulate platinum group elements which are emitted by catalytic converters of motor vehicles can accumulate in the environment as bioavailable soluble forms (34). In addition, it should be noted that platinum is difficult to extract from PM samples for ICP/MS analysis so the platinum results from our analysis are likely an underestimate of true platinum concentrations. Nonetheless, others have exposed immature and mature DC with subtoxic levels of particulate platinum group elements (34). These studies showed that platinum enhanced co-stimulatory molecule expression by DC which were then able to promote a pro-allergic IL-5 response of co-cultured T cells (34). This study supports the hypothesis that inhalable environmental particulate pollutants exhibit an adjuvant-like effect on DC and thus amplify the immune response to allergen. Thus, one can appreciate that ambient PM presents to the DC and the immune system in general, a myriad of possible activating cues. These include both biological and inorganic stimuli that collectively may all play their role in directing novel responses by DC including our current observations of dysregulated TLR and adaptor protein expression.

The lack of expression of both TLR2 and TLR4 on human monocyte-derived DC following stimulation with APM has several potential consequences. Foremost among these concerns is the potential for suppressed immunity and an inability to respond appropriately to bacterial or viral infections. We propose that suppression of TLR expression could render DC hypo-responsive to subsequent exposure to infectious agents. Others have suggested that PM may be immunosuppressive from studies in animal models (35–37). In one such study, exposure of already infected mice to inhaled airborne environmental particulate matter (PM10) worsened both the local infection as well as altered local pulmonary and systemic immunity (38). Because exacerbations of asthma or chronic obstructive pulmonary disease are usually driven by airway infections, our data suggest that one link between PM exposure and exacerbations of these conditions may be due to a relative immune suppression and predisposition to infections, although this hypothesis awaits determination. Alternatively, the diminution in expression of TLRs in response to APM could be an inherent mechanism to limit the scope of pro-inflammatory airway hyperactivity and subsequent cellular damage due to oxidative stress.

Also, in murine models of airway inflammation, it has been shown that TLR4 is required for airway hyperactivity that is seen following subchronic ozone inhalation (39). In addition, in controlled exposure experiments in human subjects, exposure to an ambient concentration of diesel exhaust particles caused airway inflammation that was characterized by activated neutrophil influx and enhanced exhalation of carbon monoxide which implicated oxidative stress being involved in the observed airway inflammation (40). Although TLRs were not studied, the authors confirmed that diesel exhaust particles provoke an inflammatory response in normal human subjects (40).

Toll-like receptors are centrally involved in allergic sensitization (41), the hygiene hypothesis (42), and in the response to ozone and other pollutants (43,44). Because PM can deliver TLR ligands, with or without viable microbes, to target cells in the respiratory tract, TLR’s mediate innate responses to noninfectious immunostimulatory materials present in the every day environment and have thus been the subject of intense scrutiny by many groups. Mutations in TLR4 are associated with endotoxin hypo-responsiveness in human subjects (45) and TLR4 deficient mice are protected from LPS induced lung inflammation (46). Recent studies indicate that TLR4 signaling can attenuate allergic inflammation in mice (45), and that the effects of TLR4 in the lung are largely mediated by hematopoietic precursors and not structural lung cells (47). This is in keeping with studies by Hunninghake et al. who showed that lung airway epithelial cells are hypo-responsive to LPS due to low TLR4 expression, but that this could be upregulated by viral infection (48).

DC express functionally diverse TLRs (e.g. TLR2, TLR3, TLR4 and TLR5) that enable DC to rapidly respond to inflammatory “danger” signals (49–51). Alterations in expression of or signaling by TLRs may expose the individual to community acquired or opportunistic infections. These are conditions favorable to the exacerbations of asthma and COPD commonly found in vulnerable populations. However, TLR-independent signals enable DC maturation via G-protein coupled receptors as well as TNF-receptors type I (p55TNFR), type II (p75TNFR) and CD40. A pathway shared by TLRs is mobilization of NF-κB that activates unique transcripts involved in DC activation (e.g. inflammatory cytokines and chemokines). While occupation of TLR4 by LPS induces activation of the NF-κB RelA: p50 pathway, occupation of CD40 by CD40 ligand induces an alternative NF-κB RelB:p52 signaling pathway and expression of novel target genes (52–54).

We believe that additional experiments are warranted that will test our proposal that TLR2 and TLR4 responses by human myeloid DC be explored as potential biomarkers for environmental particulate matter exposure. The reasons for this argument are manifold. First, engagement of TLRs is critical in the appropriate host immune responses to and sensing of infection, particularly in the context of DC (innate immunity) engaging the adaptive T cell-mediated (protective) immune response. Second, in terms of the development of allergic disease, both TLR2 and TLR4 exhibit polymorphisms in their genes that may alter the protective effect of microbial exposure as proposed in the hygiene hypothesis (55).

Thus, we believe that since dendritic cells are the most important antigen presenting cells in the immune system, any alteration in the expression or function of TLRs will have implications for DC to appropriately sense infection and mount a protective immune response against infectious microorganisms. Further, should exposure to particulate pollution promote down-regulation in the expression of either TLR2 or TLR4 in early childhood or adolescence, we would argue that this would compromise the protective effect of subsequent microbial exposures against development of subsequent allergic diseases in later life. This is a scenario defined by the hygiene hypothesis and one that is likely to be experienced by those individuals inhabiting urbanized inner cities and industrialized towns. By contrast, exposure to particulate pollution in later life may also down-regulate TLR2 and TLR4 expression with the net effect that this may dampen somewhat protective immunity in the non-allergic healthy individual but more importantly, may exacerbate the infectious disease complications of those individuals with pre-existing allergic asthma. By sampling either circulating DC or populations of DC that can be harvested and studied from samples of bronchalveolar lavage (BAL) fluid, the relative expression levels of TLRs, as defined by sensitive cellular and molecular assays, could be exploited as biomarkers of particulate pollutant effect in inner city populations.

We conclude that particulate matter exposed DC exhibit a non-classical and differential mode of activation that is phenotypically and functionally distinct from DC activated by other classical stimuli such as LPS or CD40L. Future studies will focus on the molecular and cellular mechanisms of action of particulate matter at the level of DC activation. It is anticipated that such work will confirm the potential value of measuring alterations in the expression of TLR2 and TLR4 by dendritic cells as biomarkers of airborne particulate matter effect and may open the way for targeted pharmacological therapy to interrupt the detrimental affects of particulate matter on normal innate immunity.
